# Post-traumatic intracranial pseudo-aneurysms of posterior circulation: a comprehensive review of an under-diagnosed and rare entity

**DOI:** 10.1007/s10143-021-01657-4

**Published:** 2021-10-04

**Authors:** Mauro Palmieri, Alessandro Pesce, Giuseppa Zancana, Daniele Armocida, Aniello Maiese, Carlo Cirelli, Antonio Santoro, Paola Frati, Vittorio Fineschi, Alessandro Frati

**Affiliations:** 1 Human Neuroscience Department - Neurosurgery Division “Sapienza” University, Roma, Italy; 2 Santa Maria Goretti Hospital, Neurosurgery Division, Latina, Italy; 3 Department of Anatomical, Histological, Forensic and Orthopaedic Sciences SAIMLAL – “Sapienza” University, Rome, Italy; 4 Department of Radiological, Oncological and Anatomopathological Sciences, Unit of Interventional Neuroradiology, “Sapienza” University of Rome, Umberto I University Hospital, Rome, Italy; 5 IRCCS “Neuromed”, Pozzilli, IS Italy

**Keywords:** Post-traumatic brain aneurysms, Intracranial aneurysms, Brain trauma, Vascular lesions, Complications

## Abstract

Traumatic aneurysms are rare and the total number of cases involving the posterior circulation (TIPC) is even smaller. Traumatic brain injury (TBI) may be responsible not only of rupture in brain aneurysm (BrA) pre-existing to trauma, but it has been identified also as a possible pathogenetic cause of TIPC formation in patients not affected by intracranial vascular lesions. A complete literature review was performed of all reported cases regarding rupture of BrA with SAH resulting from TIPC not previously identified at the first radiological screening. A representative case of a left posterior inferior cerebellar artery (PICA) pseudo-aneurysm caused by left vertebral artery’s dissection is reported. We show a unique complete collection of all 34 cases. Despite their rarity, TIPCs are associated with a significant morbidity and mortality rate, as high as 40–60%. Of the 22 patients with good neurological status (64.7%), we did not notice a significant correlation with regard to the location of the aneurysm, type of treatment, or clinical onset. Early recognition of a pseudo-aneurysm and adequate treatment seem to be the most important prognostic factor for these patients. Despite their rarity, TIPCs are associated with a significant morbidity and mortality rate. A TIPC should be suspected in case of delayed deterioration in head‐injured patient and should be investigated with angiography. Conservative management is worsened by poor prognosis and the goal of treatment is to exclude the aneurysm from circulation with surgical or endovascular methods as soon as possible.

## Introduction

Brain aneurysms (BrA) represent the most common intracranial vascular condition, with an incidence of 1–2% [33]. BrA that develops following head injuries presents a rare and unique challenge both during the diagnostic and surgical treatment phases [17, 40]. Traumatic intracranial aneurysms are rare, comprising 1% or less of all cerebral aneurysms and the total number of cases involving the posterior circulation is even smaller.

A spontaneous rupture of BrA addresses for the 80% of non-traumatic cases of subarachnoid hemorrhage (SAH) [18] with a reported case fatality rate that ranges between 25 and 50% (where several risk factors have been identified in high blood pressure, kidney disorders, alcohol consumption, and arterial walls’ infections [13, 34]). BrA rupture resulting from traumatic brain injury (TBI) represents a well-note but less common occurrence [13]. TBI may be responsible not only for rupture in BrA pre-existing to trauma, but it has also been identified as a possible pathogenetic cause of pseudo-aneurysms’ formation in patients not affected by intracranial vascular lesions before the traumatic event, where this occurrence is less common in the posterior intracranial circulation. Traumatic intracranial pseudo-aneurysm of the posterior circulation (TIPC) is most frequently reported in the first three decades of life. TIPCs have been reported in association with both closed and penetrating trauma [44, 47]; even though it is described as a rare occurrence, the formation of these particular types of pseudo-aneurysms that the dissection of cervical arteries might cause [28] is due to high-speed TBI [10].

There are quite a few case reports on this topic; however, larger studies, let alone prospective studies, are lacking, so the real incidence is not known, what are the risk factors, and why they are entities so complicated to diagnose compared to their supratentorial counterparts.

In the present paper, a complete literature review was performed of all reported cases in which a rupture of BrA with SAH results from TIPC was not previously identified at the first radiological screening. Also, we report a representative case from our department experience of a left posterior inferior cerebellar artery (PICA) pseudo-aneurysm caused by the left vertebral artery’s dissection.

## Material and methods

The study was conducted following preferred reporting items for systematic reviews.

The English literature was systematically investigated using MEDLINE, the NIH Library, PubMed, and Google Scholar. The last search date was June 15, 2021.

Search terms included: “post-traumatic” or “traumatic” in combination with “intracranial” or “brain aneurysm.” Searches were limited to human studies and no limits regarding language, and we excluded a period of publication before 1978. Backward citation tracking was applied to identify articles not retrieved by electronic searches.

We have fully focused our attention on TIPC as it is less common and described in reported cases of post-traumatic aneurysms and because, we believe, they are more subtle from the point of view of symptom onset and diagnostic imaging.

### Selection criteria

Two independent authors conducted the selection of abstracts for full review based on predefined inclusion and exclusion criteria. Studies were eligible if they reported original data on the case of posterior post-traumatic BrA. Studies excluded were as follows: (a) papers regarding aneurysm located in other locations than the posterior circulation; (b) case report related to penetrating traumas or with (c) with incomplete data; (d) paper presenting a re-analysis of subpopulations already included in other studies; were commentaries or review articles summarizing the results of the previous series; (e) year of publishing was also included to understand a possible year of experience/improvement of the technologic setup, and (f) the articles do not report or specify the diagnostic and therapeutic measures adopted.

**Fig. 1:**
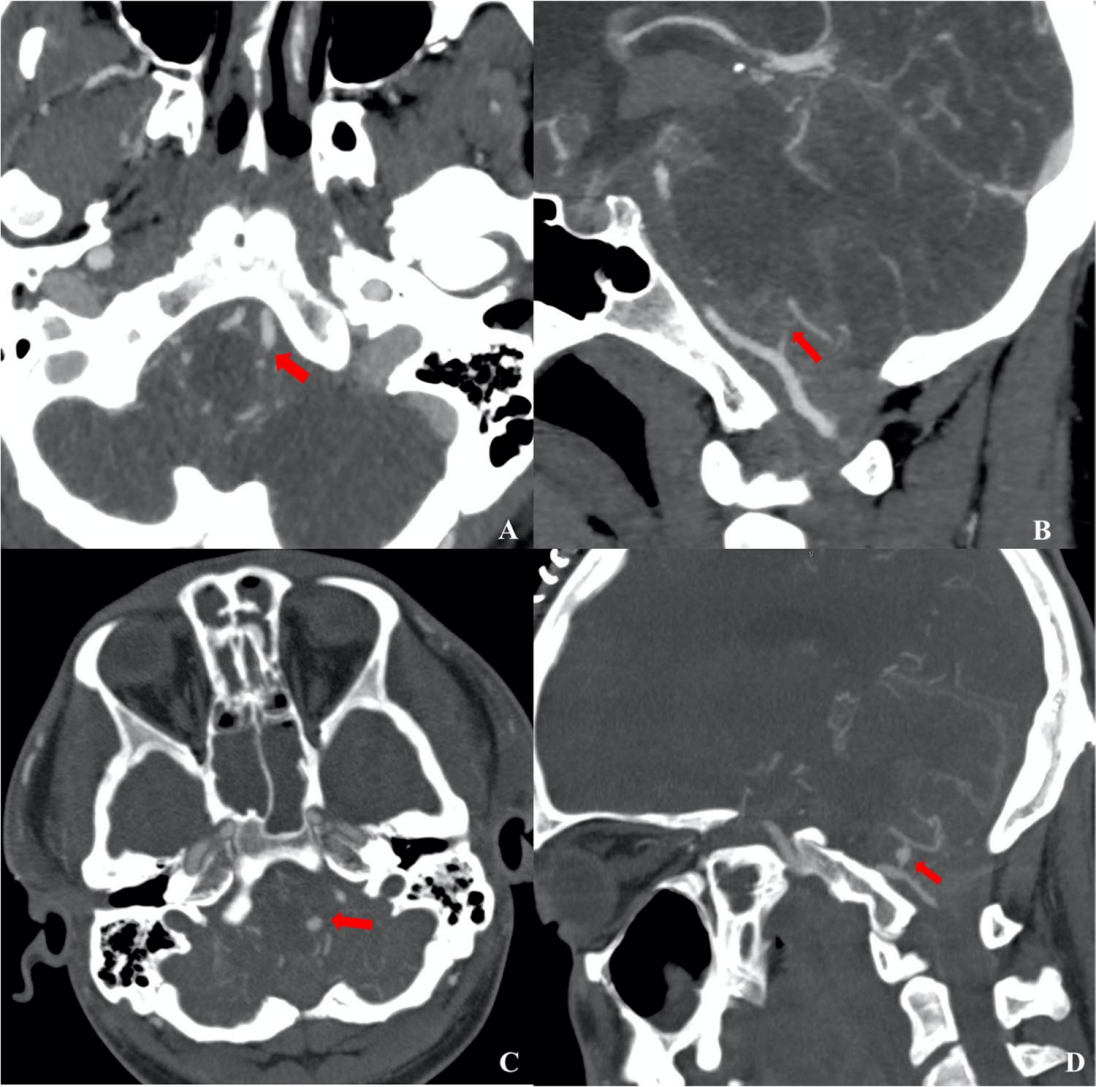
**A** Axial and **B** sagittal scans o the first angio-CT and the same scans in **C** and **D** 9 days after the trauma. The arrows highlight the site of the left PICA where the post-traumatic aneurysm has formed

Each author reviewed the abstracts independently and generated a list of studies to retrieve for full-text review (Fig. 1).

### Representative case

A 26-year-old male with a negative medical history was led to the Emergency Department because of a progressively worsening state of consciousness after severe physical aggression with concomitant TBI and consequent fall to the ground with further brain injury. At admission, blood analysis showed strong positivity for cannabinoids and alcohol. The neurological onset measured with the Glasgow coma scale (GCS) at admission was 8. Therefore, a brain computed tomography (CT) was performed, and it disclosed the presence of a subarachnoid hemorrhage (SAH) located in Sylvian, prepontine, premedullary, and interpeduncular cisterns bilaterally with diffuse intraventricular hemorrhage (Fisher 4). A brain angio-CT scan was immediately performed, which did not disclose the presence of vascular lesions in cervical and intracranial arteries (Fig. 1). A bilateral frontoparietal decompressive craniectomy was performed with concomitant positioning of an external ventricular drainage. After the surgical intervention, the patient was moved to the intensive care unit (ICU). After 24 h, a brain CT was performed, which showed a reduction of cerebral edema, absence of hydrocephalus, and SAH volume stability. Pharmacological sedation was suspended on the seventh post-operative day.

**Fig. 2:**
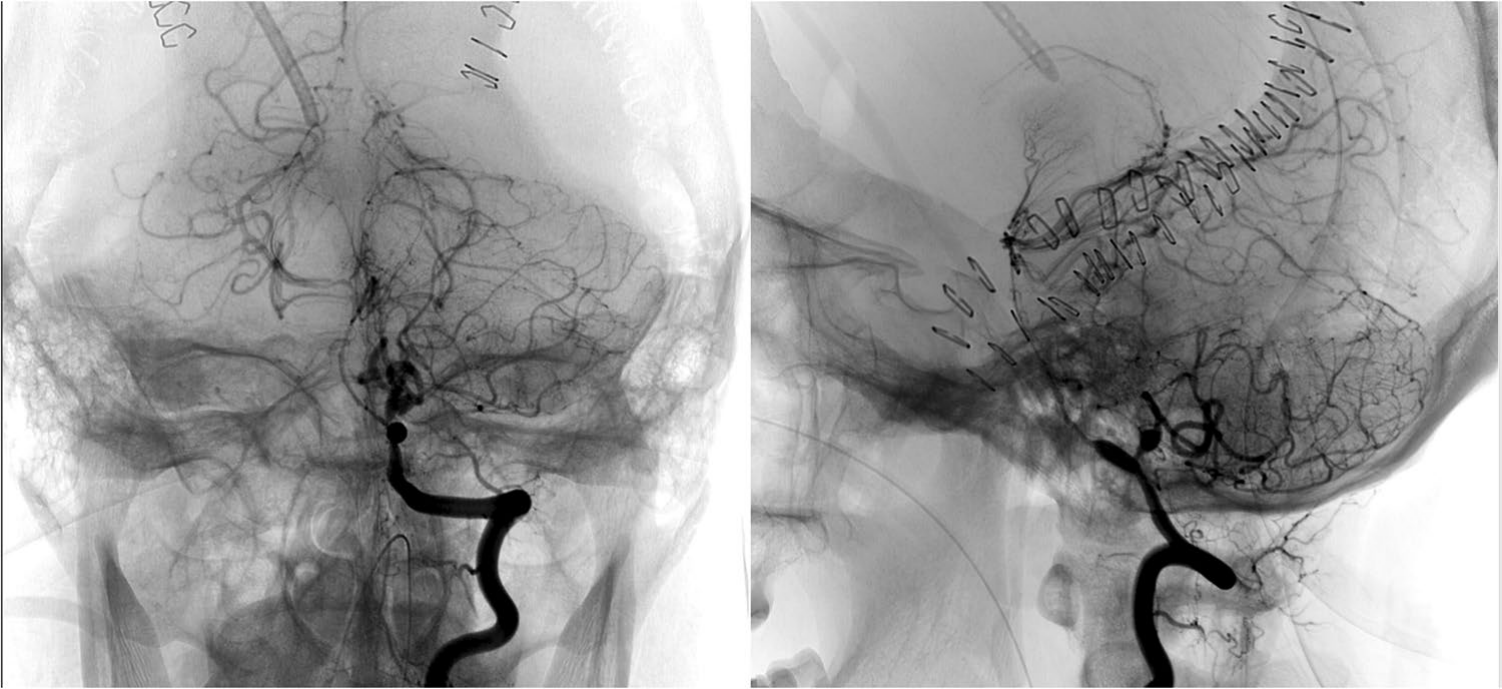
DSA frames showing the presence of the post-traumatic aneurysm and the concomitant presence of left V4 tract of the vertebral artery wall’s dissection

Nine days after the trauma, the patient suddenly became unresponsive, the GCS dropped to 3, and a brain CT was performed immediately, highlighting an increased volume of the SAH volume in the peribulbar cistern the hemorrhage in the third and fourth ventricles (Fig. 1). Therefore, a brain angio-CT was performed, which showed massive vasospasm of all intracranial arteries and ectasia of the left V4 tract of vertebral artery of 11 mm with stenosis of the cranial segments of the artery, the origin of the left postero-inferior cerebellar artery (PICA), and the V4 tract of the right vertebral artery (VA). Moreover, a sacciform aneurysmal dilation with a maximum dimension of 3.6 × 4.5 mm at the origin of the left PICA was highlighted, with anterolateral projection and signs of sac’s rupture (Fig. 2). Consequentially, a DSA was performed, which confirmed the diffused arterial vasospasm, the presence of the aneurysm, and the ectasia of V4 due to dissection of the vertebral artery (Fig. 2). Since decompressive craniectomy has been performed already and given the good functioning of the external ventricular drainage, the diffuse cerebral vasospasm, and the critical clinical and neurological conditions, no surgical or endovascular procedures were proposed. After this episode, the patient’s hospitalization continued in ICU.

In the following days, brain CT and a brain MRA were performed, which showed the unchanged dimension of both PICA aneurysm and V4 dissection, confirmed by a cerebral DSA executed after 1 month.

**Fig. 3:**
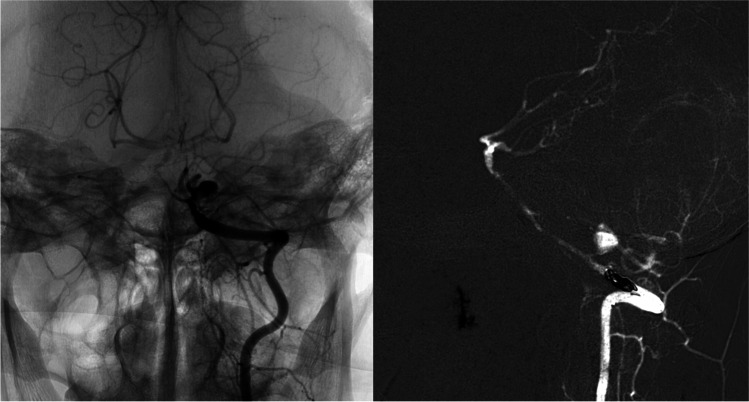
DSA frames showing the result of the embolization of the left vertebral artery, carried out 3 months after the trauma

In the following 3 months, the radiological follow-up showed the consequences of diffuse cerebral ischemia; hence, no modifications of the aneurysmal dilation were highlighted (Fig. 3) until a selective DSA for the study of the left PICA was performed. The exam, performed 4 months after the sudden post-operative neurological worsening, showed an increase of the aneurysm’s dimension, from 3.6 × 4.5 mm to 9.0 mm. Therefore, endovascular embolization of the left V4 tract of the left vertebral artery was performed through coiling (Fig. 3). One month later, the patient died due to sudden respiratory insufficiency.

## Results

The main search returned a total of 831 papers, including a total of 477 patients. To this initial cohort, the exclusion, as mentioned earlier, criteria were applied, accordingly eliminating 801 papers for a total of 33 patients. To this cohort of patients, the personal experience of a unique case of post-traumatic BrA was added.

A data extraction sheet was designed to extract all the necessary information visible in Table 1. The data were subsequently verified between the two authors. The following details were extracted: authors and year of publication, sex, age, type of trauma, neurological status expressed as GCS, reported onset symptoms, intracranial pathological signs reported at first CT-scan, symptoms occurred during the observation phase for which the patient performed a second CT control, the result of first DSA, the time occurred to the first diagnosis of pseudo-aneurysm, localization, type of treatment and outcome.

**Table 1:** Patient’s demographics with abbreviations

Authors	Sex	Age	Trauma modality	GCS	Reported symptoms at onset	Onset CT	Delayed symptoms	Second CT	First DSA	Time to diagnosis	Localization	Treatment	Outcome
Meguro et. al., 1985 [24]	M	54	Auto vehicle accident	14	Rousable, no movement right upper limb	Hydrocephalus, IVH	\	\	Negative	6 days	Right PICA	Surgery	Good
Quattrocchi et. al., 1990 [37]	M	26	Fall	4	Coma	SAH, IVH, hydrocephalus	\	\	\	\	Left SCA	\	Dead
Morard et. al., 1991 [26]	M	31	Fall	3	Coma	SAH, IVH	\	Persistent SAH	\	10 days	Right PICA	Surgery	Good
Quintana et. al., 1996 [38]	M	27	Assault	13	Stupor and left-side hemiparesis	Hydrocephalus, IVH	\	Contusion	\	3 days	BA	Endovascular	Good
Proust et. al., 1997 [35]	F	22	Bicycle accident	15	Asymptomatic	\	Headache	Nodular neoformation of the right free edge of the tentorium	\	3 months	Right SCA	Surgery	Good
Loevner et. al., 1998 [21]	M	7	Fall	15	Asymptomatic	Negative	Left third nerve palsy and a sudden change in mental status	Midbrain hematoma	\	9 months	BA	Conservative	Good
Ramesh et. al., 1998 [40]	M	34	Fall	3	Coma	SAH	\	\	\	\	Left VA	\	Dead
Schittek, 1999 [42]	M	22	Falling object	15	Pulsatile mass in the right lateral neck triangle	\	\	\	\	5 years	Right VA	Surgery	Good
Schuster et. al., 1999 [43]	M	22	Assault	3	Coma	SAH, IVH	\	\	Pseudo-aneurysm	\	Right PICA	Surgery	Good
	M	16	Falling object	3	Coma	SAH, IVH	\	\	Negative	72 h	Right PICA	Surgery	Good
Kibayashi et. al., 2000 [15]	M	20	Assault	3	Coma	SAH, IVH	\	\	\	\	Left PICA	No	Dead
Connor et. al., 2001 [6]	F	12	Auto vehicle accident	4	Coma	SAH, IVH, SDH	Proptosis	Enhancing lesion	\	20 days	BA	Endovascular	Good
Nishioka et. al., 2002 [29]	M	20	Falling object	3	Coma	SAH, IVH	\	\	\	12 days	Right PICA	No	Dead
	F	33	Assault	14	Headache and stiff neck	SAH, IVH	\	\	Pseudo-aneurysm	\	Left PICA	Surgery	Good
Ashley Jr et. al., 2005 [2]	M	37	Falling object	7	Coma	Negative	\	\	Pseudo-aneurysm	\	Left VA	Endovascular	Good
Moron et. al., 2005 [27]	M	6	Fall	14	Headache	Brain contusions	Headache	Thrombosis	2 days: pseudo-aneurysm	15 days	Left PCA	Spontaneous and complete thrombosis	Good
Kaiser et. al., 2008 [14]	M	40	Assault	\	\	SAH, IVH	\	\	Pseudo-aneurysm	\	Left VA	Endovascular	Dead
McElroy et. al., 2008 [23]	M	62	Falling object	7	Soporose	SAH	\	New SAH	Negative	3 weeks	BA	Endovascular	Good
Binning et. al., 2009 [3]	M	15	Assault	7	Soporose	SAH and hydrocephalus	\	Abnormality near vertebral artery	Negative	21 months	Left PICA	Surgery and stent	Good
Ong et. al., 2009 [30]	M	3	Fall	\	Asymptomatic	\	Unable to be roused from sleep (15 days than trauma)	SAH with hydrocephalus	\	15 days	Left SCA	Endovascular	Dead
Coulter et al., 2011 [7]	F	20	Auto vehicle accident	14	Headache	SAH	\	\	Pseudo-aneurysm	\	Right VA	Endovascular	Good
Paiva et. al., 2011 [31]	M	31	Assault	11	Soporose	SAH and hydrocephalus	GCS 7	\	\	\	Left SCA	Endovascular	Good
Li et. al., 2013 [20]	M	34	Motorcycle accident	7	Soporose	Brain contusions	Epistaxis	\	\	28 days	BA	Endovascular	Good
Griauzde et. al., 2014 [11]	F	7	Motorcycle accident	3	Coma	SAH	\	\	\	5 months	BA	Endovascular	Good
Kim et. al., 2014 [16]	M	51	Fall	12	Confusion	SAH	GCS 3	New SAH	Extravasation on the basilar artery	1 day	BA (top)	Endovascular	Dead
Purgina et. al., 2014 [36]	M	22	Assault	14	Confusion	SAH, IVH, hydrocephalus	GCS 3	New SAH	Negative	9 days	Left PICA	No	Dead
Huh et. al., 2015 [12]	M	9	Auto vehicle accident	9	Soporose	SAH, IVH	Semicoma	New IVH	\	15 days	Right VA	Endovascular	Dead
Desouza et. al., 2016 [9]	M	20	Fault	15	Asymptomatic	SAH, IVH, hydrocephalus	Cardiac arrest		Negative	11 days	VA	\	Dead
	M	21	Assault	14	Confusion	SAH and hydrocephalus	GCS 10	\	\	1 day	Left PICA	Endovascular	Good
Lee et. al., 2016 [19]	F	55	Fall	3	Coma	SAH	Sixth nerve palsy	\	Negative	1 month	Right PICA	Surgery	Good
Sujijantarat et. al., 2017 [45]	M	14	Motorcycle accident	6	Coma	SAH	\	\	Pseudo-aneurysm	\	SCA	Surgery	Vegetative
Sami et. al., 2018 [41]	F	24	Motorcycle accident	14	Loss of vision in the right eye	\	\	\	Pseudo-aneurysm	\	Right PCA	Endovascular	Good
Ciochon et. al., 2020 [5]	M	30	Bicycle accident	15	Asymptomatic	EDH and SDH	GCS 3	Increased SAH	\	9 days	Left PCA	Endovascular	Good
Our Case (2019)	M	26	Assault	8	Soporose	SAH	GCS 3	Increased SAH	Pseudo-aneurysm	9 days	Right PICA	Endovascular	Dead

Abbreviations: *IVH* intraventricular hemorrhage, *SAH* sub-arachnoid hemorrhage, *SDH* sub-dura hemorrhage, *EDH* epidural hemorrhage, *BA* basilar artery, *PICA* postero-inferior cerebellar artery, *VA* vertebral artery, *PCA* posterior cerebral artery, *SCA* superior cerebellar artery

The patients in total, including our representative case, are 34 (27 males and 7 females), with an average age of 25.67. The most common traumatic cause reported concerns about vehicle accidents (29.4%), assaults (29.4%), and accidental falls (26.4%).

Patients at the time of the first neurologic evaluation most frequently have severe neurological status with 12 patients in a coma (35.3%) and in 47% of cases GCS less than 8.

However, we found wide variability in the presentation of the initial symptoms, also highlighting asymptomatic patients with accidental or mild headache (26.47%).

As can be seen from the table, in no case is the presence of aneurysm reported at the first CT scan radiological control time. Of all the cases analyzed, no patient underwent intracranial DSA at admission to the emergency unit.

Patients repeated a new radiological control as a follow up (in 55.9%) or at the onset of new neurological disorders (44.1%). The in-depth diagnostic study with DSA occurred in 16 patients (47.05%) of cases with time elapsed from the traumatic event varying between 24 h and 5 months. Notably, only in 8 patients of our collection, the pseudo-aneurysmatic formation was present at the time of the first angiographic control.

Once the pseudo-aneurysmatic formation with a new DSA was identified, the most common location was the PICA in 12/34 patients (35.3%), followed by the location in the basilar artery (BA) and VA in 20.5% (7 BA and 7 VA cases), superior cerebellar artery (SCA, 6 cases, 17.6%), and posterior cerebral artery (PCA, 2 cases, 5.9%) respectively. The treatment choice is also highly variable in this case, with 16 patients treated by endovascular route (47%) and 10 treated with a surgical procedure (29.4%). We find only one reported case treated with a combined endovascular and neurosurgical approach. On 3 reports, the procedure is not reported, while for 4 patients, the choice for conservative treatment is reported for clinical severity or spontaneous and complete thrombosis (2 patients, 5.88% of cases).

Despite their rarity, according to literature, TIPCs are associated with a significant morbidity and mortality rate, as high as 40–60% [44.47]. As an outcome measure, we identified as GOOD status patients discharged with an undamaged neurological examination or with mild disorders that did not impact their performance status. Of the 22 patients with good neurological status (64.7%), we did not notice a significant correlation between the aneurysm location, type of treatment, or clinical onset. However, early recognition of a pseudo-aneurysm and adequate treatment seem to be the most important prognostic factor for this particular category of patients where the small collection does not allow for more in-depth statistical evaluations.

A TIPC should be suspected in case of delayed deterioration in a head‐injured patient and should be investigated with angiography [1, 4, 5, 22, 25, 44, 47]. Conservative management is worsened by poor prognosis [25, 28, 34].

## Discussion

The post-traumatic BrA represents a rare condition, with a reported incidence that ranges from 3.2–3.6% to 5.7%, and they account for less than 1% of all BrA [25]. According to histological features, post-traumatic aneurysms are classified in [8]:

- True aneurysms: the lesion affects tunica intima of the arterial wall, with variable initial involvement of the internal elastic lamina and tunica media, while tunica externa is preserved. This condition weakens the different layers of the vessel’s wall, creating intraluminal hemodynamic changes that determine the formation of the aneurysm, with the possibility of partial rupture and consequent repair.
- False aneurysms (or pseudo-aneurysms): they are generally associated with penetrant traumas, which causes transmural lesions of the arterial wall and consequent formation of an extra-parietal hematoma. The bleeding caused by the rupture of these types of BrA starts immediately after the trauma and the risk of second bleeding is extremely high since it may occur even after the resolution of surrounding hematoma and cerebral edema [36].
- Mixed aneurysms: these are real BrA that, due to the formation of a transmural hematoma after rupture, develop a false lumen.

Post-traumatic BrA is most commonly located in the anterior circulation, even though seldom cases of lesions located in correspondence of VA and BAs have been reported [17]. This particular kind of BrA usually forms near fractures or where the artery is closely connected to the dura mater [38]. Moreover, when an extracranial artery enters the dura, the external elastic lamina disappears; hence, the arterial wall is weaker and more susceptible to rupture [39].

PICA aneurysms are rare, with a general incidence of 2.8% [32], but if we consider only the series of PITC, they become the most frequent site, and they are most commonly located at the origin of the artery. Saccular aneurysms are usually located at the distal portion of the artery, while BrA originating from dissection are encountered in the proximal tract, associated with parent vessel’s ectasia and concurrent distal or proximal stenosis [46], exactly like the case described in the present paper.

Since cases of SAH following post-traumatic BrAs’ rupture located in the posterior circulation are rare, only seldom reports can be found in current Literature (Table 1). Shuster et al. [43] reported 3 cases of post-traumatic SAH, 2 of those related to the posterior tract of the PICA, which occurred shortly after closed-head TBI, suggesting that even moderate traumas can cause rupture of PICA aneurysms. According to these authors, this might be related to particular anatomical variants of PICA and predisposing vascular disease [43]. Moreover, they suggest that the presence of persistent hydrocephalus and symptomatic vasospasm in their cases is evidence that corroborates the traumatic etiopathogenesis of the rupture since these particular clinical features are reported in only the 5–12% of spontaneous ruptures of PICA’s aneurysms [43]. Nishioka et al. [29] reported 2 analog cases, proposing the brainstem’s sudden movement on the sagittal plane with acceleration-deceleration of the cerebellum as the possible pathogenetic cause of the rupture. Further evidence is retrieved from the case of a young male involved in a scuffle [15], in which it is suggested that the PICA’s rupture might be caused by the transitory occlusion of the homolateral vertebral artery due to rotatory movements of the neck that may be associated to a blood flow inversion from the basilar artery to the occluded vertebral artery, causing PICA’s rupture at its origin.

Even though that the only possible way to exclude the pre-existence of the aneurysm would have been to perform a radiological exam before the trauma, the negativity for vascular lesion highlighted by the brain CTA at admission suggests that the first SAH was probably due to dissecting damage of left vertebral artery caused by the trauma. Moreover, the entity of the physical aggression and the consequent further brain injury and the positivity for cannabinoids and alcohol could explain the severity of the neurological state at admission. This particular lesion led to the formation of the PICA’s aneurysm, which dimensions progressively grew in the days following the trauma, along with the increase of entity of the dissection located at the V4 tract of the left vertebral artery.

Most aneurysms manifest angiographically in 2–3 weeks with an average of 20 days [1, 17, 33] and often this examination is performed to investigate the reasons for an incomprehensible clinical worsening or for late post-hemorrhagic control, and these data are also confirmed to examine the pseudo-aneurysm of the posterior circulation. It is well known that arterial vasospasm can hide the eventual presence of BrA in the context of diffuse SAH [22]. However, an important consideration is relevant in this particular case: the brain CTA performed at admission did not show a suggestive presence of arterial vasospasm, while the CTA and, even more, the cerebral DSA executed when the post-operative neurological worsening occurred showed the existence of the aneurysm and the dissection of the vertebral artery despite the presence of severe cerebral vasospasm. This evidence supports the thesis that the aneurysm most probably was not formed before the trauma or, at least, its dimensions were not notable for being identified through radiological exams at admission.

Moreover, an important limitation given by the difficulty of understanding this high rate of misdiagnosis at the beginning of the pathological process of aneurysmal formation is that what kind of DSA was done is almost never reported (if images from 2 directions, images from 4 directions or 3D); however, the quality and techniques of DSA should be discussed, if an increase of angiograms is recommended.

So, the diagnosis of TIPC requires a high index of suspicion; anytime a head‐injured patient presents a delayed neurological deterioration, it should be promptly submitted to cerebral DSA [47].

In conclusion, we affirm that a follow‐up angiogram should be obtained even if the initial one was negative. The second DSA should be performed at least 3 weeks after the trauma, and it could be argued that a third angiogram should be performed 6–12 weeks after injury [4, 34, 47].

## Conclusions

Despite their rarity, TIPCs are associated with a significant morbidity and mortality rate. A TIPC should be suspected in case of delayed deterioration in a head‐injured patient with concomitant pronounced SAH and should be investigated with angiography after performing a brain CTA in first place. A poor prognosis worsens conservative management, and the goal of treatment is to exclude the aneurysm from circulation with surgical or endovascular methods as soon as possible.

## Data Availability

Not applicable.
